# Carbon Monoxide Regulates Macrophage Differentiation and Polarization toward the M2 Phenotype through Upregulation of Heme Oxygenase 1

**DOI:** 10.3390/cells10123444

**Published:** 2021-12-07

**Authors:** In-Soon Kang, Rang-Ie Kim, Chaekyun Kim

**Affiliations:** 1 Laboratory of Leukocyte Signaling Research, Department of Pharmacology, College of Medicine, Inha University, Incheon 22212, Korea; round001@hanmail.net (I.-S.K.); good_angei@naver.com (R.-I.K.); 2 BK21 Program in Biomedical Science & Engineering, Inha University, Incheon 22212, Korea; 3 Convergent Research Center for Metabolism and Immunoregulation, Inha University, Incheon 22212, Korea

**Keywords:** carbon monoxide, heme oxygenase, CO-releasing molecule-3, M1/M2 macrophage, polarization

## Abstract

Carbon monoxide (CO) is generated by heme oxygenase (HO), and HO-1 is highly induced in monocytes and macrophages upon stimulation. Monocytes differentiate into macrophages, including pro-inflammatory (M1) and anti-inflammatory (M2) cells, in response to environmental signals. The present study investigated whether CO modulates macrophage differentiation and polarization, by applying the CO-releasing molecule-3 (CORM-3). Results showed that murine bone marrow cells are differentiated into macrophages by CORM-3 in the presence of macrophage colony-stimulating factor. CORM-3 increases expressions of macrophage markers, including F4/80 and CD11b, and alters the cell morphology into elongated spindle-shaped cells, which is a typical morphology of M2 cells. CORM-3 upregulates the expressions of genes and molecules involved in M2 polarization and M2 phenotype markers, such as STAT6, PPARγ, Ym1, Fizz1, arginase-1, and IL-10. However, exposure to CORM-3 inhibits the iNOS expression, suggesting that CO enhances macrophage differentiation and polarization toward M2. Increased HO-1 expression is observed in differentiated macrophages, and CORM-3 further increases this expression. Hemin, an HO-1 inducer, results in increased macrophage differentiation, whereas the HO-1 inhibitor zinc protoporphyrin IX inhibits differentiation. In addition, CORM-3 increases the proportion of macrophages in peritoneal exudate cells and enhances the expression of HO-1 and arginase-1 but inhibits iNOS. Taken together, these results suggest that the abundantly produced CO in activated macrophages enhances proliferation, differentiation, and polarization toward M2. It will probably help clear apoptotic cells, resolve inflammation, and promote wound healing and tissue remodeling.

## 1. Introduction

Heme oxygenase (HO) produces carbon monoxide (CO) in most cell types during the catabolism of heme, along with biliverdin/bilirubin and free iron [1,2]. Among the HO isoforms, HO-1 is the inducible isoform that provides protection against oxidative stress, whereas HO-2 and HO-3 are constitutively expressed. HO-1 expression is regulated by the redox-sensitive transcription factor, the nuclear factor (erythroid-derived 2)-like 2 (Nrf2). It is highly induced in macrophages when exposed to oxidative stress and inflammation and also during differentiation [3,4]. HO-derived CO acts as a potent antioxidant and anti-inflammatory mediator and protects cells by inhibiting cell death [5,6,7].

Monocytes differentiate into macrophages after migrating out of the blood vessel and are polarized into various subsets by multiple signals in all tissues [8]. The monocyte-to-macrophage differentiation involves extensive transcriptome changes that are tightly controlled by various signaling molecules and transcriptional regulators. Macrophages can be grouped broadly as subsets of classically activated (M1) or alternatively activated (M2) cells. M2 are further divided into subsets of M2a, M2b, M2c, and M2d [9,10]. M1 are induced in the inflammatory environment and are associated with immunity to bacteria and intracellular pathogens, whereas M2 are arisen by Th2 responses, such as helminth immunity, asthma, and allergy [8,11]. Although M1 and M2 share some common characteristics, the remarkable plasticity between M1 and M2 is evident by their response to the local tissue environment [12]. M1 polarization occurs in the presence of interferon (IFN)-γ, granulocyte-macrophage colony-stimulating factor (GM-CSF), and lipopolysaccharide (LPS) via signal transducer and activator of transcription 1 (STAT1), interferon regulatory factor 5 (IRF5), or nuclear factor kappa-light-chain-enhancer of activated B cells (NF-κB), whereas fungal and helminth infections, interleukin (IL)-4, IL-10, IL-13, and tumor growth factor (TGF)-β skew macrophages toward M2 via STAT6, peroxisome proliferator-activated receptor γ (PPARγ), PPARδ, or Jumonji domain-containing protein D3 [8,13]. Moreover, the plasticity of macrophages is proven by re-polarization of already differentiated macrophages when M2 are exposed to M1 signals or vice versa [14,15].

M1 have a unique ability to metabolize arginine to nitric oxide (NO), which damages and kills pathogens through production of peroxynitrite (ONOO^−^), whereas M2 metabolize arginine to ornithine, which is involved in tissue repair processes [16,17]. Thus, M1 and M2 express high levels of inducible nitric oxide synthase (iNOS) and arginase (Arg)-1, respectively. In addition, M1 express high levels of CD68, CD80, CD86, IL-1R, Toll-like receptor (TLR) 2 and 4, and suppressor of cytokine signaling 3, whereas M2 express high levels of CD163, CD206, chitinase-3-like protein (Chi3l3 or Ym1/2), and found in inflammatory zone 1 (Fizz1) [8]. In addition, M1 are associated with the ability to secrete pro-inflammatory mediators, such as IL-1β, IL-12, tumor necrosis factor (TNF)-α, reactive oxygen species (ROS), and NO, whereas M2 produce high levels of anti-inflammatory mediators including TGF-β and IL-10. Accordingly, phenotypical differences of macrophage are associated with different roles in diseases, e.g., M1 play a pro-inflammatory role, whereas M2 play an anti-inflammatory role and resolve inflammation [18,19,20].

The present study investigated the effects of CO on macrophage differentiation and polarization using a CO-releasing molecule, tricarbonylchloro(glycinato)ruthenium(II) (CORM-3) [21]. CORM-3 enhances the macrophage colony-stimulating factor (M-CSF)-derived differentiation of murine bone marrow cells (BMCs) toward M2 showing upregulation of genes and molecules involved in M2 polarization and inhibition of iNOS expression. CORM-3 also increases Nrf2 and HO-1 expressions. Hemin or zinc protoporphyrin IX (ZnPP) treatment increases or abates the macrophage differentiation, respectively. In addition, macrophages obtained after intraperitoneal injection of CORM-3 into thioglycollate-induced peritonitis reveal the M2 phenotype.

## 2. Materials and Methods

### 2.1. Reagents and Antibodies

Human recombinant RANKL, murine M-CSF (also called CSF-1), IFN-γ, IL-4, GM-CSF, and TNF-α were procured from PeproTech (London, UK). Minimum essential medium (α-MEM), phosphate buffered saline (PBS), penicillin, and streptomycin were purchased from HyClone (Logan, UT, USA). Oligonucleotides and SYBR Green PCR Master Mix were bought from Bioneer (Daejeon, Korea) and Toyobo (Osaka, Japan), respectively. Antibodies against the following antigens were used in this study: HO-1 (Enzo Life Sciences, Farmingdale, NY, USA), Nrf2 (Santa Cruz Biotechnology, Santa Cruz, CA, USA), peroxiredoxin 1 (Prx1) (AbFrontier, Seoul, Korea), and anti-mouse CD16/CD32 (BD Bioscience, San Jose, CA, USA). Horseradish peroxidase-conjugated mouse- and rabbit-IgG antibodies were purchased from BD Pharmingen (San Diego, CA, USA). Other chemicals were purchased from Sigma-Aldrich (St. Louis, MO, USA) unless otherwise stated.

### 2.2. Mice

C57BL/6J mice procured from Jackson Laboratory (Bar Harbor, ME, USA) were housed under pathogen-free conditions at the animal facility of Inha University (Incheon, Korea). The study was conducted according to the guidelines for the Care and Use of Laboratory Animals published by the National Institute of Health (NIH, USA 2013) and the ARRIVE guidelines and approved by the Inha University-Institutional Animal Care and Use Committee (INHA-IACUC) (approval Number INHA-161214-465).

### 2.3. Differentiation of Bone Marrow Cells

BMCs were obtained from the femur, tibia, and pelvis bones [22]. Briefly, bones were flushed with α-MEM and centrifuged at 500× *g* for 5 min at 10 °C and the red blood cells were lysed with lysis buffer containing 155 mM NH_4_Cl, 10 mM KHCO_3_, and 0.1 mM EDTA. BMCs were cultured overnight in α-MEM supplemented with 10% FBS and 10 ng/mL M-CSF. Non-adherent BMCs were collected the next day and cultured continuously with 30 ng/mL M-CSF for 8–12 days, replenishing the medium every 3 days, to induce differentiation into macrophages. M-CSF is an essential growth factor for the survival, differentiation, and activation of macrophages. The M-CSF receptor (c-fms) is present only in the monocyte/macrophage lineage. Cells were treated with 0–500 µM CORM-3 on day 5, followed by 5 days of incubation. In addition, cells were also treated with the HO inducer and inhibitor (20 µM hemin and 20 µM ZnPP, respectively) in the presence of CORM-3 on day 5.

### 2.4. Preparation of Peritoneal Macrophages

Mice were intraperitoneally administered 3% Brewer thioglycollate medium; 24 h later, they were administered saline (control group) or 20 and 40 mg/kg CORM-3. A total of 72 h after thioglycollate injection, the mice were sacrificed, and the peritoneal cavity was lavaged twice with cold PBS [22]. Total peritoneal exudate cells were counted, and cells were subjected to cytospin using the Shandon-cytospin 3 (Astmoor, England). Cells were stained with Diff-Quik (Dade Behring, Malvern, PA, USA), and differential cell counts of the peritoneal exudates were determined under a microscope (Olympus, Tokyo, Japan) equipped with Leica optical microscope camera (Wetzlar, Germany).

### 2.5. Flow Cytometry

Flow cytometry analysis was performed to assess the expression of F4/80 and CD11b. Cells were fixed with 4% formaldehyde in PBS for 10 min on ice, suspended in PBS containing 0.1% bovine serum albumin (BSA), and incubated with an anti-mouse CD16/CD32 for 10 min to block the Fc receptor. Cells were incubated with fluorescein isothiocyanate (FITC)-conjugated antibody against F4/80, phycoerythrin (PE)-conjugated antibody against CD11b, or relevant isotype control antibody (eBioscience, San Diego, CA, USA) for 30 min on ice. There were no significant differences in the staining intensity of pre-fixing staining versus post-fixing staining using these antibodies. After washing, stained cells were acquired by flow cytometry (FACSCalibur, BD Biosciences) and analyzed using the FlowJo v10.8 software (Treestar, Ashland, OR, USA).

### 2.6. Quantitative Reverse Transcription Polymerase Chain Reaction (qRT-PCR)

Total RNA was extracted from macrophages using a total RNA isolation reagent (Invitrogen, Carlsbad, CA, USA) and subjected to reverse transcription according to the manufacturer’s protocol (Takara Bio, Tokyo, Japan). The qRT-PCR was subsequently performed with the Applied Biosystems StepOne instrument (Foster City, CA, USA) using SYBR Green PCR Master Mix (Toyobo). The primers used for qRT-PCR are listed in Table 1. Relative gene expression was normalized to glyceraldehyde 3-phosphate dehydrogenase (GAPDH).

### 2.7. Western Blotting

Cell lysates were prepared, and total protein was measured by applying the bicinchoninic acid protein kit from Pierce (Rockford, IL, USA) using BSA as a standard. Total protein (20–30 µg) was separated by sodium dodecyl sulfate-polyacrylamide gel electrophoresis, followed by transfer onto polyvinylidene fluoride membranes (Millipore, Bedford, MA, USA). The blots were probed with specific antibodies and developed using the enhanced chemiluminescent kit (Thermo Fisher Scientific, Rockford, IL, USA). Integrated densitometry was used to determine the intensity of scanned films using the ImageJ 1.48v software (NIH, Bethesda, MD, USA).

### 2.8. Statistical Analyses

Two-tailed Student’s *t*-test was performed using Microsoft Excel (Redmond, WA, USA). Multiple comparisons were performed using one-way analysis of variance (ANOVA) with Tukey test using SigmaPlot v11 software (Systat software Inc., San Jose, CA, USA). Data are expressed as the mean ± standard deviation (SD), and *p* < 0.05 is considered statistically significant.

## 3. Results

### 3.1. CO Stimulates Macrophage Differentiation

**Figure 1:**
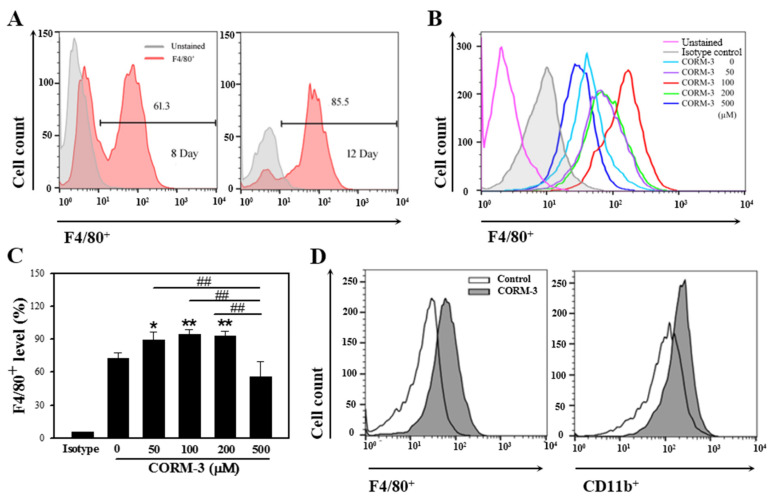
CORM-3 enhances macrophage differentiation. Murine BMCs were differentiated with 30 ng/mL M-CSF for 8–12 days to induce macrophage differentiation and cells were acquired on FACSCalibur. (**A**) Representative flow cytometry of F4/80-positive cells on days 8 and 12 (n = 3). (**B**,**C**) BMCs were treated with 0–500 μM CORM-3 in the presence of 30 ng/mL M-CSF on day 5 and incubated for 5 days. F4/80-positive cells were analyzed on FACSCalibur (n = 3). Results were presented as mean ± SD, * *p* < 0.05, and ** *p* < 0.01 compared to 0 μM CORM-3, and ^##^ *p* < 0.01 between groups compared (one-way ANOVA). (**D**) BMCs were treated with 100 μM CORM-3, and F4/80-positive and CD11b-positive cells were analyzed on FACSCalibur (n = 34).

Monocytes originate from common myeloid precursors in the bone marrow and undergo differentiation into macrophages or dendritic cells after migrating out of the capillary blood vessel. In the present study, murine BMCs were differentiated into macrophages in the presence of M-CSF; around 60% of cells were F4/80-positive on day 8 and more than 80% of cells were F4/80-positive on days 10–12 (Figure 1A). The exogenous administration of CO mimics the effects of HO-1 activation, and, therefore, the BMCs were treated with CORM-3. Exposure to CORM-3 resulted in increased expressions of macrophage-specific markers, F4/80 and CD11b, as compared to the non-treated control, thus indicating that CO enhances differentiation of BMCs toward macrophages (Figure 1B–D). The highest increase was observed at 100 μM CORM-3 concentration, and we, therefore, treated BMCs with 100 μM CORM-3 for the subsequent experiments. CORM-3 had no effect on cell viability at concentrations lower than 200 μM as measured using the MTT assay (Figure S1).

### 3.2. CO Enhances Macrophage Polarization toward the M2 Phenotype

**Figure 2:**
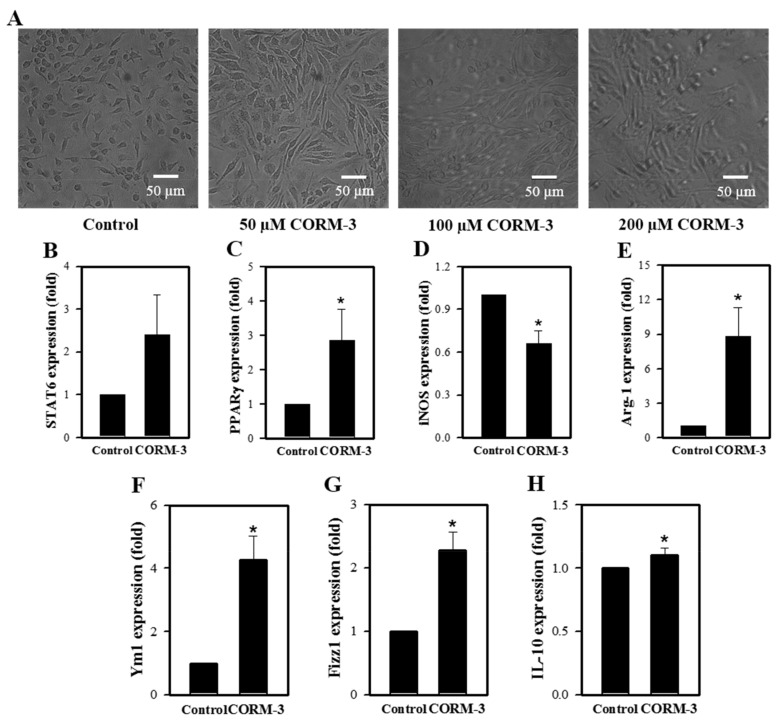
CORM-3 increases genes and molecules involved in M2 polarization but decreases iNOS expression. Murine BMCs were treated with CORM-3 on day 5 in the presence of 30 ng/mL M-CSF and incubated for 5 days to induce macrophage differentiation. (**A**) Representative pictures of cells treated with 0–200 μM CORM-3 (n = 3). (**B**–**H**) The mRNA expression levels of STAT6, PPARγ, iNOS, Arg-1, Ym1, Fizz1, and IL-10 were determined by qRT-PCR (n = 3). GAPDH was used as an internal reference. Results are presented as mean ± SD, * *p* < 0.05 compared to control (Student’s *t*-test).

Untreated macrophages (M0) are predominantly small and roundish with a noteworthy proportion having an amoeboid morphology. M1 are characterized by an enlarged amoeboid cell shape with roundish cell bodies, and M2 are comprised of a heterogeneous cell population, which includes spindeloid cells and multinucleated giant cells [23]. Although rather heterogeneous, CORM-3 treatment alters the cell morphology to elongated spindle-shaped cells, a typical functional phenotype of the M2 cells (Figure 2A). To further investigate the effects of CO on M1/M2 polarization, the mRNA expressions of genes and markers of M1 and M2, such as STAT6, PPARγ, iNOS, Arg-1, Ym1, Fizz1, and IL-10, were determined by qRT-PCR. Expression of the M1 marker iNOS was decreased in response to CORM-3; however, genes and molecules related to M2 showed increased expressions (Figure 2B–H), confirming that CO enhances macrophage differentiation toward the M2 phenotype.

### 3.3. Different Ligands Differentially Stimulate M1/M2 Polarization

**Figure 3:**
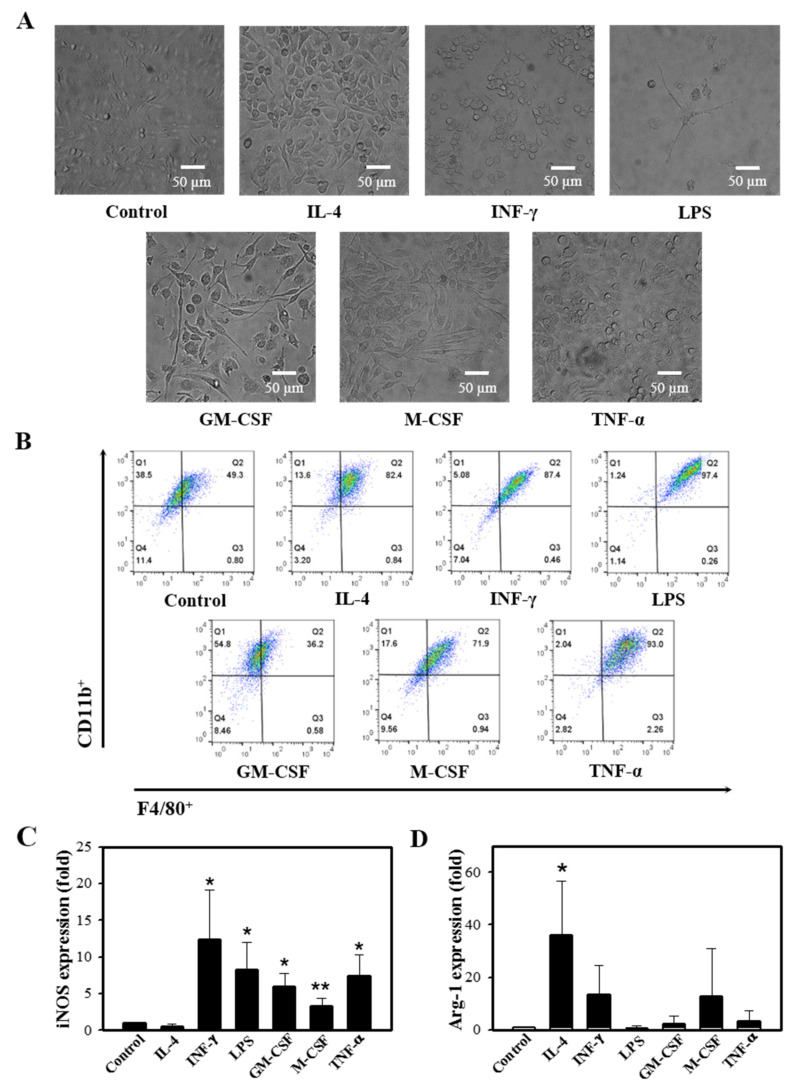
Different ligands induce M1/M2 polarization. Murine BMCs were treated with a variety of stimuli, such as 10 ng/mL IL-4, 50 ng/mL IFN-γ, 10 µg/mL LPS, 10 ng/mL GM-CSF, 30 ng/mL M-CSF, and 10 ng/mL TNF-α in the presence of 30 ng/mL M-CSF on day 5, followed by 5 days of incubation. (**A**) Cell images after 10 days of differentiation were acquired (n = 4). (**B**) Representative flow cytometry of cells in response to stimuli, X axis: F4/80^+^, Y axis: CD11b^+^ (n = 3). (**C**,**D**) Relative gene expressions of iNOS and Arg-1 by the M1/M2 modulating stimuli were determined by qRT-PCR (n = 3). Results are presented as mean ± SD; * *p* < 0.05 and ** *p* < 0.01 compared to control (Student’s *t*-test).

It is well documented that macrophages alter their functional phenotype in response to the microenvironmental influences [9,24]. BMCs were treated with a variety of stimuli, including IFN-γ, GM-CSF, M-CSF, LPS, TNF-α, or IL-4, which are known to direct the M1/M2 polarization. The stimulation resulted in increased macrophage differentiation (Figure 3A,B), and treatment with IFN-γ, TNF-α, M-CSF, GM-CSF, and LPS upregulated the iNOS expression, whereas IL-4 treatment resulted in increased Arg-1 expression (Figure 3C).

### 3.4. CO Increases HO-1 Expression in Macrophages

**Figure 4:**
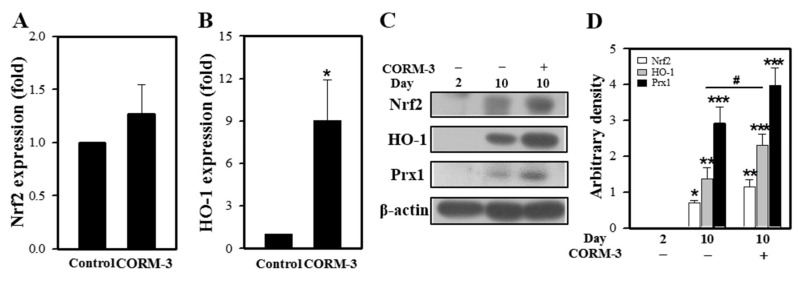
CORM-3 increases Nrf2 and HO-1 expression. Murine BMCs were treated with 100 μM CORM-3 on day 5 and incubated for 5 days to induce macrophage differentiation. (**A**,**B**) Gene expression of Nrf2 and HO-1 was measured using qRT-PCR (n = 3). (**C**) The protein levels of Nrf2 (n = 3), HO-1 (n = 5), and Prx1 (n = 4) in differentiated macrophages were determined by immunoblotting and (**D**) the relative densities were calculated as the ratio to β-actin. Results are presented as mean ± SD; * *p* < 0.05, ** *p* < 0.01, and *** *p* < 0.001 compared to control, and ^#^ *p* < 0.05 between groups compared with Student’s *t*-test for (**A**,**B**) or one-way ANOVA for (**D**).

Since CO induces HO-1 expression through Nrf2 activation [25], we determined the effect of CORM-3 on HO-1 expression. Expressions of Nrf2 and HO-1 mRNA were increased by CORM-3 treatment (Figure 4A,B). HO-1 protein expression was negligible in BMCs at day 2 and increased in differentiated macrophages at day 10; treatment with CORM-3 further increased the HO-1 expression and Prx1 expressions (Figure 4C,D), indicating that exogenous CO upregulates Nrf2 and HO-1 and the increased HO-1 produces more CO, which further stimulates macrophage differentiation and M2 polarization. In addition, inactive CORM-3 (iCORM-3) was prepared by dissolving CORM-3 in PBS and allowing liberation of CO for 24 h at 20–23 °C [26]. Treatment cells with iCORM-3 did not affect HO-1 expression (data not shown), reflecting that the HO-1 induction observed with CORM-3 was a result of released CO.

### 3.5. Endogenous CO Enhances Macrophage Differentiation and Inhibition of HO-1 Suppresses the Differentiation

**Figure 5:**
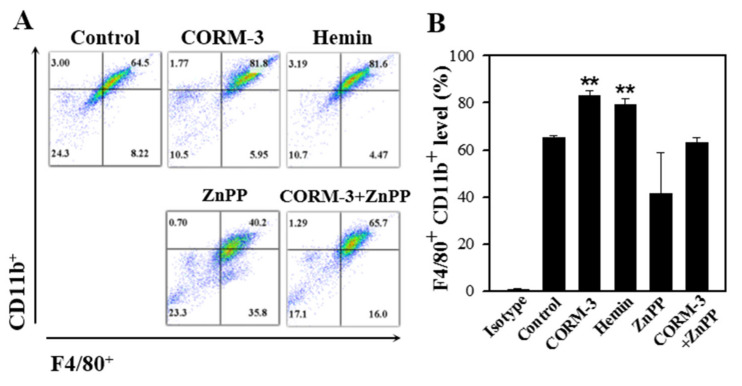
HO-1 inducer enhances macrophage differentiation and HO-1 inhibitor suppresses the differentiation. Murine BMCs were treated with 20 μM hemin, 20 μM ZnPP, or 20 μM ZnPP with 100 μM CORM-3 on day 5 and incubated for 5 days to induce macrophage differentiation. (**A**) Cells were fixed and stained with antibodies F4/80-FITC and CD11b-PE. Cells were acquired on FACSCalibur, X axis: F4/80^+^, Y axis: CD11b^+^. (**B**) F4/80-positive and CD11b-positive cells were gated based on the isotype control. Results are presented as mean ± SD (n = 3 except n = 2 for CORM-3+ZnPP); ** *p* < 0.01 compared to control (one-way ANOVA).

To determine the effect of endogenous CO on macrophage differentiation, BMCs were treated with either an HO-1 inducer (hemin) or an HO-1 inhibitor (ZnPP). The concentrations of hemin and ZnPP used in this experiment did not affect cell viability as measured using the MTT assay (Figure S2). Treatment with hemin increased the F4/80-positive and CD11b-positive cell populations, but ZnPP suppressed the macrophage differentiation (Figure 5). Moreover, combined treatment of CORM-3 with ZnPP suppressed the macrophage differentiation, suggesting that macrophage differentiation is tightly regulated by the CO availability. Along with the results shown in Figure 4, these results indicate that HO-1 expression increases during macrophage differentiation, and the increased HO-1 produces more CO, which further stimulates macrophage differentiation.

### 3.6. CO Enhances M2 Polarization of Peritoneal Macrophages

**Figure 6:**
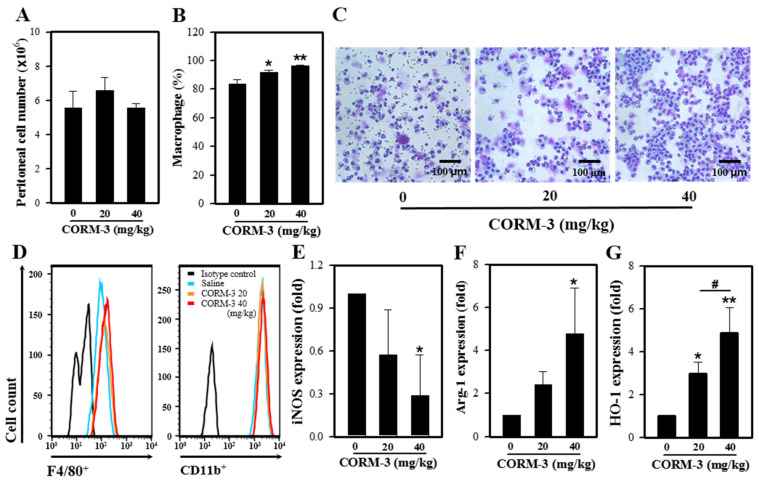
CO enhances M2 polarization in vivo. Mice were injected with 3% thioglycollate followed by CORM-3, and peritoneal exudate cells were collected. (**A**) The number of total peritoneal exudate cells was counted. (**B**) The proportion of macrophages in peritoneal exudates was determined after Diff-Quik staining, and (**C**) the representative images of peritoneal exudate cells. (**D**) Peritoneal exudate cells were stained with antibodies F4/80-FITC and CD11b-PE and analyzed using FACSCalibur. (**E**–**G**) The mRNA expressions of iNOS, Arg-1, and HO-1 were determined by qRT-PCR. Results are presented as mean ± SD (n = 3); * *p* < 0.05 and ** *p* < 0.01 compared to control and ^#^ *p* < 0.05 between groups compared (one-way ANOVA).

We earlier showed that CORM-3 inhibits iNOS expression but increases the expressions of Arg-1 and HO-1 in vitro. In an effort to determine whether CORM-3 affects the expression of these genes in vivo, we administered CORM-3 into the thioglycollate-treated peritoneum and collected the peritoneal exudate cells. Total peritoneal exudate cell counts were similar between control and CORM-3 groups (control, 5.6 × 10^6^ cells vs. 40 mg/kg CORM-3, 5.6 × 10^6^ cells) (Figure 6A). Although CORM-3 increased the proportion of macrophages in peritoneal exudate cells compared to control (control, 84% vs. 40 mg/kg CORM-3, 97%) (Figure 6A–C), the F4/80-positive and CD11b-positive cell populations determined by flow cytometry were similar between groups (Figure 6D). Similar to the in vitro results, mRNA expression of iNOS was decreased, whereas the expressions of Arg-1 and HO-1 were increased in peritoneal exudate cells (Figure 6E–G), confirming that CO enhances macrophage differentiation toward M2.

## 4. Discussion

Hematopoietic cells express high levels of HO-1, which influences a lineage commitment in pluripotent stem cells and maturation of hematopoietic cells [27]; in particular, HO-1 is abundantly expressed in monocytes/macrophages [28]. When macrophages overexpress HO-1 or are exposed to CO, the proinflammatory response is inhibited whereas the anti-inflammatory response is enhanced [6,29]. Therefore, we assumed that CO plays a role in macrophage differentiation and polarization. Yamamoto-Oka et al. [30] showed that CORM-3 promotes progression of the M2 phenotype, as evidenced by the increased expressions of CD206 and Ym1. However, CORM-3 also upregulated expression of the M1 marker iNOS in naïve rat alveolar macrophages. To elucidate these conflicting results, we investigated the effect of CO on macrophage differentiation and polarization. We found that CO enhances macrophage differentiation and increases expressions of the M2 markers but decreases the M1 marker iNOS.

In general, the expression level of iNOS is applied to define M1 polarization, and Ym1, Fizz1, or Arg-1 are mouse M2 markers with no human homologs [31]. The known signaling molecules involved in M2 polarization are STAT6, IRF4, Krüppel-like factor-4, NF-κB p50, PPARγ, hypoxia inducible factor-2α, IL-21, bone morphogenetic protein-7, fatty acid binding protein 4, and Liver X receptor alpha. Arg-1, a distinct hallmark of M2 along with Mrc1, Fizz1, and PPARγ, is induced by IL-4 and IL-3 via STAT6 [13]. In the present study, CORM-3 exposure resulted in increased macrophage differentiation and polarization toward M2, which was evidenced by the increase of F4/80-positive and CD11b-positive cells, as well as increased STAT6, PPARγ, Arg-1, Ym1, Fizz1, and IL-10 and decreased iNOS expressions. Collectively, these results are consistent with the report that HO-1 induction in macrophages functionally switches these cells to the M2 phenotype [32].

It was previously reported that exogenous CO induces HO-1 expression via Nrf2 activation in liver cells and endothelial cells [25]. Thus, we examined whether CORM-3 could activate Nrf2 and induce HO-1 in macrophages. HO-1 expression was induced during macrophage differentiation, and exposure to CORM-3 further increased the expression (Figure 4). Moreover, endogenously produced CO by hemin enhanced the F4/80-positive and CD11b-positive cell populations, whereas inhibition of HO-1 by ZnPP suppressed the differentiation. These findings are similar to the HO-1 knockout phenotype or Nrf2 knockdown during macrophage differentiation [33]. The myeloid-specific HO-1 deletion in mice exhibited increased mRNA expressions of the M1 markers, but decreased expressions of the M2 markers, suggesting that HO-1 deletion promotes M1 polarization. Accordingly, HO-1 deletion displayed increased hepatic damages, while overexpression of HO-1 exhibited the opposite in mice. The higher HO-1 levels showed lower M1 markers’ expressions, together with decreased hepatocellular damage and improved outcomes in humans [33]. Moreover, lack of Nrf2 in macrophages showed upregulation of the M1 markers and down-regulation of the M2 markers [34], indicating that Nrf2 inhibits M1 polarization while promoting M2 polarization.

These results are similar to our in vivo findings, wherein increased expression of HO-1 was observed in thioglycollate-induced peritoneal macrophages, along with decreased iNOS and increased Arg-1 expressions. Thioglycollate medium has been used to induce an inflammatory response in the peritoneal cavity [35,36]. Thioglycollate-induced peritoneal macrophages express the immature myeloid lineage cell markers Ly-6C and ER-MP58, indicating that they are not fully differentiated but possess the possibility of an activation change [36]. Macrophages are divided into M1 and M2 in response to different stimuli in vitro but are stimulus dependent and have mixed states of M0, M1, and M2 in vivo. In this study, CORM-3 significantly increased the proportion of macrophages in the peritoneal cavity (Figure 6B), suggesting that CO can enhance macrophage maturation of immature peritoneal macrophages. Moreover, the decreased iNOS and increased Arg-1 expressions in CORM-3-treated groups represent that CO can direct the change into M2 cells in vivo.

In summary, HO-1/CO stimulates macrophage differentiation and polarization toward the M2 phenotype. Exposure to CO differentiates BMCs toward the M2 morphology and increases gene expressions and signaling molecules of the M2 cells. However, since the analyses performed in this study are focused on the morphological phenotype and expression of M1/M2 markers, functional analyses will be required to confirm the observed phenotypes in future studies. Moreover, Nrf2 and HO-1 expressions are increased in the presence of CORM-3, subsequently resulting in increased CO production, which induces further HO-1 expression. The endogenous CO also increases macrophage differentiation, which is suppressed in the presence of CO inhibitor. These findings correlate with the polarization toward M2 in thioglycollate-induced peritoneal macrophages. Taken together, our findings suggest that HO-1/CO is critical for macrophage differentiation. CO produced in excess by macrophages during inflammation induces HO-1, which further produces CO, which redirects infiltrating macrophages toward M2.

## Figures and Tables

**Table 1:** List of qRT-PCR primer sequences.

Gene	Forward Primer	Reverse Primer
*Arg-1*	CTG AGA GAT TCA AGG CAA GAG G	GAA CGC GCT ATC TTA CCC CAG
*Fizz1*	CTG CCC TGC TGG GAT GAC T	CAT CAT ATC AAA GCT GGG TTC TCC
*HO-1*	AAG CCG AGA ATG CTG AGT TCA	GCC GTG TAG ATA TGG TAC AAG GA
*IL-10*	GCT CTT ACT GAC TGG CAT GAG	CGC AGC TCT AGG AGC ATG TG
*iNOS*	GTT CTC AGC CCA ACA ATA CAA GA	GTG GAC GGG TCG ATG TCA C
*Nrf2*	TCT CCT CGC TGG AAA AAG AA	ATT TCG TGT CGG TCG TGT AA
*PPAR* *γ*	TCT TCC ATC ACG GAG AGG TC	GAT GCA CTG CCT ATG AGC AC
*STAT6*	CTG GGG TGG TTT CCT CTT G	TGC CCG GTC TCA CCT AAC TA
*Ym1*	CAA GTT GAA GGC TCA GTG GCT C	CAA ATC ATT GTG TAA AGC TCC TCT C
*GAPDH*	CCT TCC GTG TTC CTA CCC C	CCC AAG ATG CCC TTC AGT

## Data Availability

The data presented in this study are included in the article and also available on request from the corresponding author.

## Supplementary Materials

The following are available online at https://www.mdpi.com/article/10.3390/cells10123444/s1. Figure S1: Effect of CORM-3 on cell viability, Figure S2: Effect of hemin and ZnPP on cell viability, Figure S3: CORM-3 increases Nrf2 expression.
