# Supplementary figures and images for: Carbon Monoxide Regulates Macrophage Differentiation and Polarization toward the M2 Phenotype through Upregulation of Heme Oxygenase 1
Source: Cells. 2021 Dec 7;10(12):3444. doi: 10.3390/cells10123444 (PMC8700076; doi:10.3390/cells10123444)

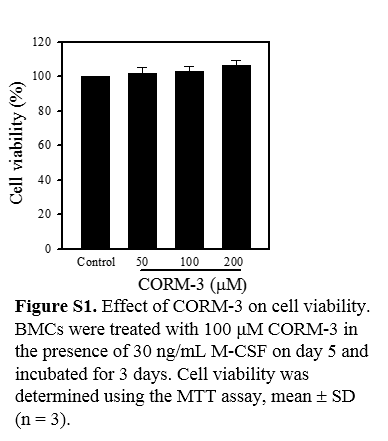

Supplement: Supplementary file 1 [file cells-10-03444-s001.zip › Figure S1.tif]

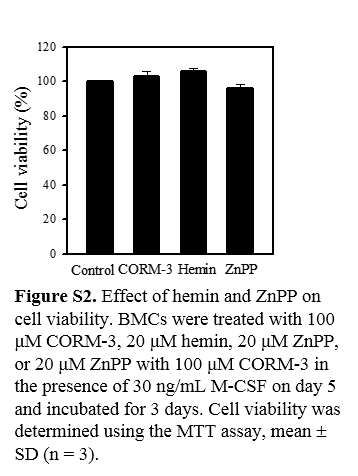

Supplement: Supplementary file 1 [file cells-10-03444-s001.zip › Figure S2.tif]

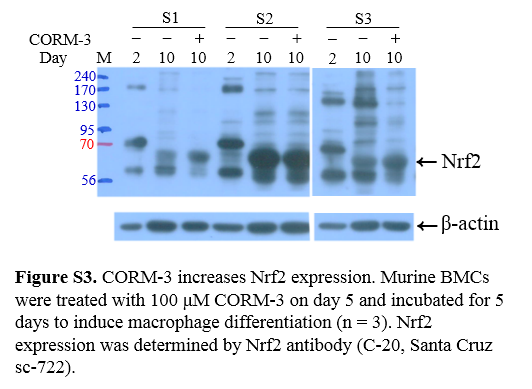

Supplement: Supplementary file 1 [file cells-10-03444-s001.zip › Figure S3.tif]
